# Supplementary material for: Promotion of Ru or Ni on Alumina Catalysts with a Basic Metal for CO2 Hydrogenation: Effect of the Type of Metal (Na, K, Ba)
Source: Nanomaterials (Basel). 2022 Mar 23;12(7):1052. doi: 10.3390/nano12071052 (PMC9000749; doi:10.3390/nano12071052)
Supplement: Supplementary file 1 [file nanomaterials-12-01052-s001.zip › nanomaterials-1652287-supplementary.pdf]

nanomaterials

MDPI

Supplementary Material

Promotion of Ru or Ni on Alumina Catalysts with a Basic Metal for CO_2 Hydrogenation: Effect of the Type of Metal (Na, K, Ba)

Enrique García-Bordejé [1,*], Ana Belén Dongil [2], José M. Conesa [2,3], Antonio Guerrero-Ruiz [3,4] and Inmaculada Rodríguez-Ramos [2,4]

[1] Department of Chemical Processes and Nanotechnology, Instituto de Carboquímica (ICB-CSIC), Miguel Luesma Castán 4, 50018 Zaragoza, Spain
[2] Institute of Catalysis and Petrochemistry, CSIC, 28049 Madrid, Spain; a.dongil@csic.es (A.B.D.); jmconesa@ccia.uned.es (J.M.C.); irodriguez@icp.csic.es (I.R.-R.)
[3] Departamento de Química Inorgánica y Química Técnica, UNED, Av. de Esparta s/n, 28232 Las Rozas de Madrid (Madrid, Spain), aguerrero@ccia.uned.es
[4] Grupo de Diseño y Aplicación de Catalizadores Heterogéneos, Unidad Asociada UNED-CSIC (ICP), 28049 Madrid, Spain
* Correspondence: jegarcia@icb.csic.es; Tel.: +34-976-733-977; Fax: +34-976-733-318

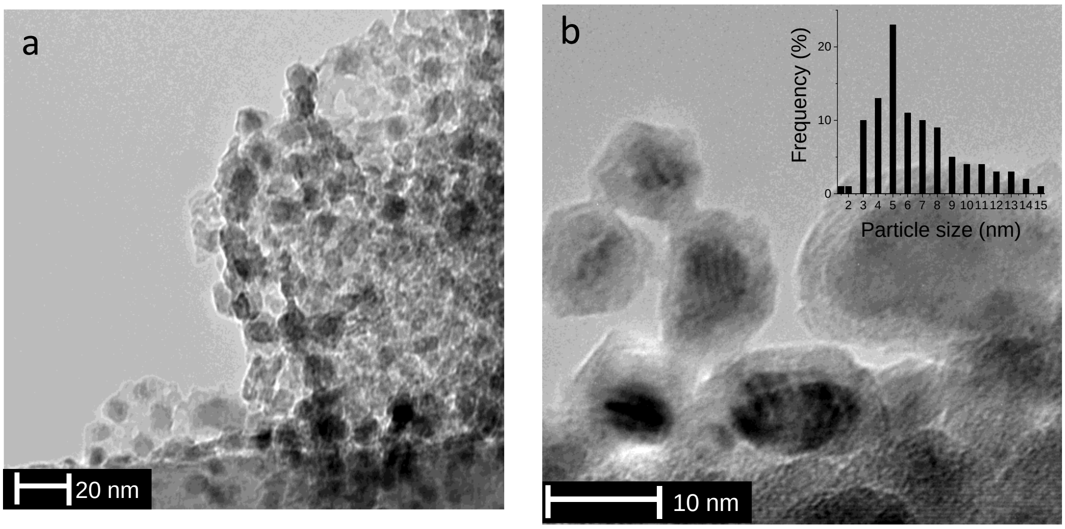

Figure S1. Representative high-resolution TEM images at two magnifications (20 nm (**a**) and 10 nm (**b**)) and particle size distribution (inset in (**b**)).

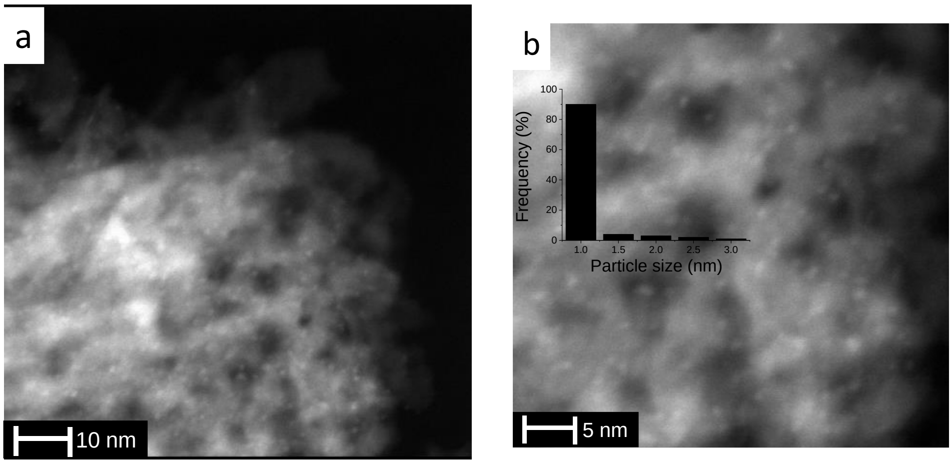

Figure S2. Representative high-resolution TEM images at two magnifications (10 nm (**a**) and 5 nm (**b**)) and particle size distribution (inset in (**b**)).

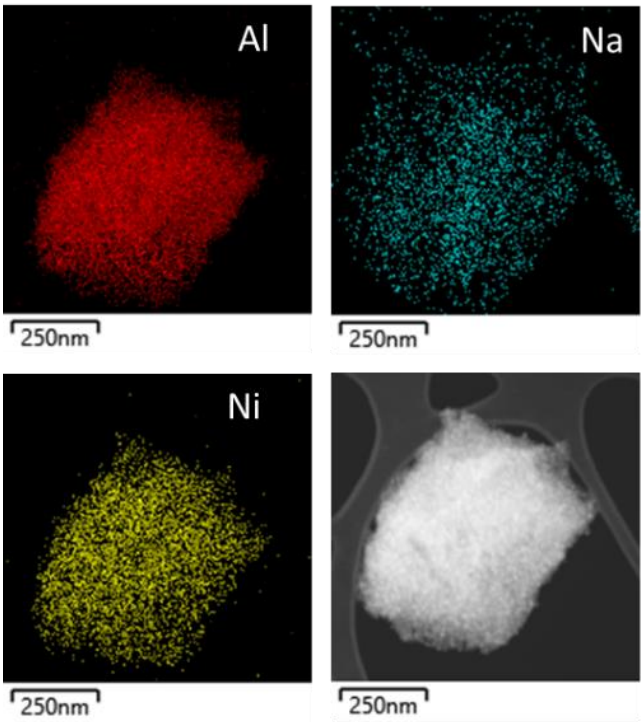

Figure S3. Representative XEDS-mapping in STEM mode for (10%Na)15%Ni/Al.

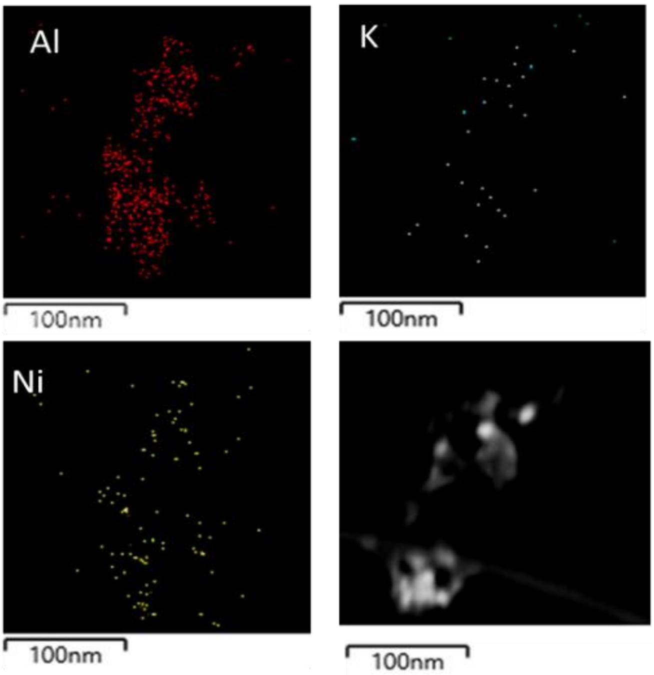

Figure S4. Representative XEDS-mapping in STEM mode for (10%K)15%Ni/Al.

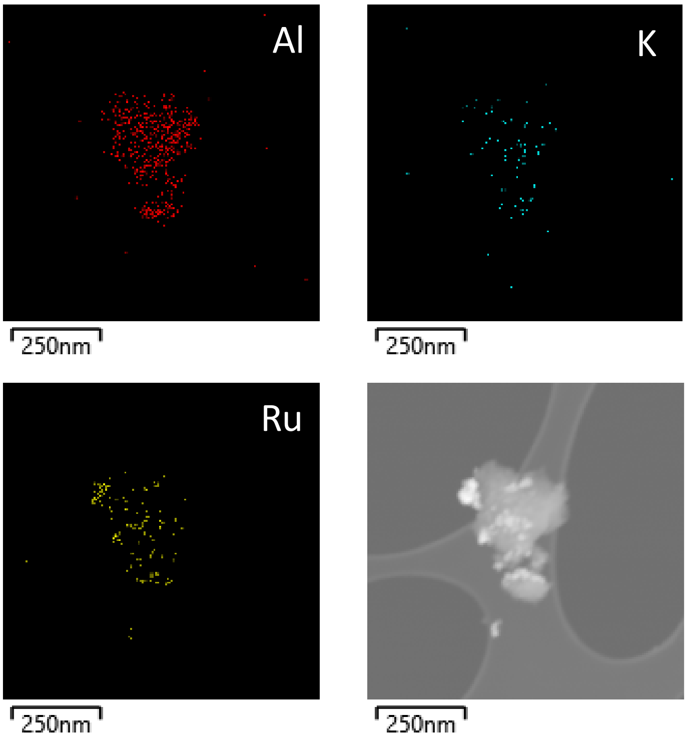

Figure S5. Representative XEDS-mapping in STEM mode for (3%Ru)10%K/Al.

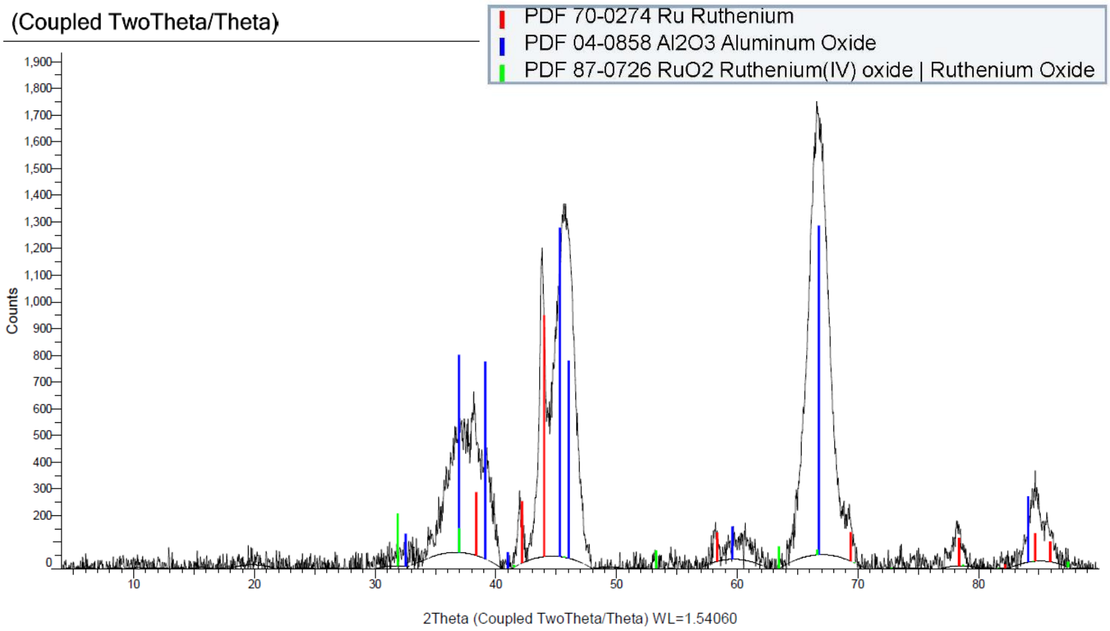

Figure S6. XRD diffractogram of as- synthesised 3%Ru/Al_2O_3 and assignment pattern of the components.

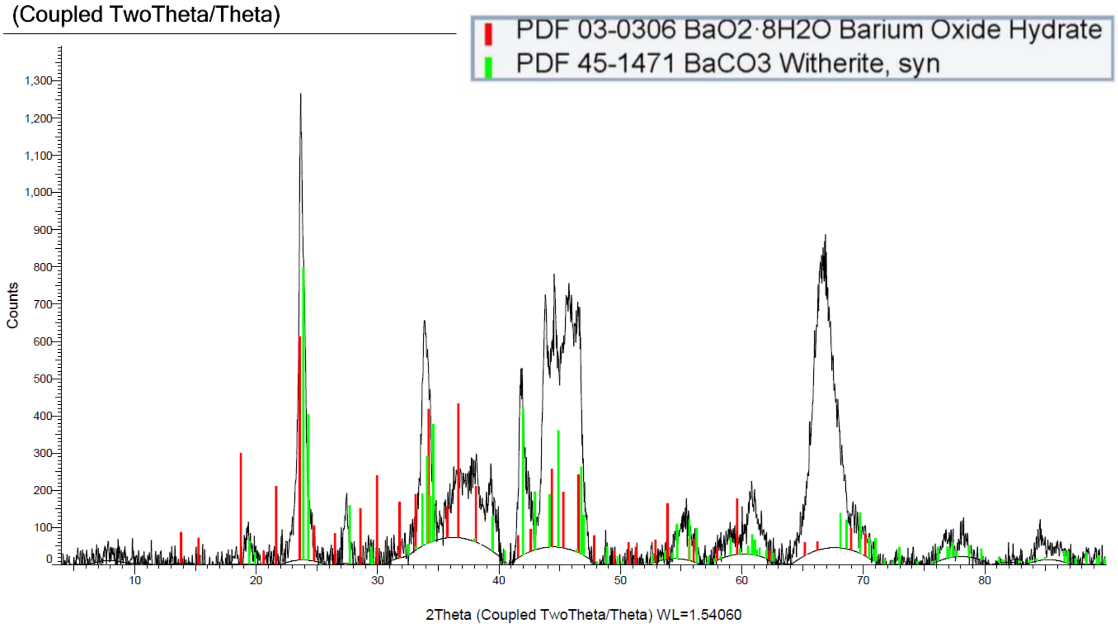

Figure S7. XRD diffractogram of as- synthesised (3%Ru)10%Ba/Al_2O_3 and assignment pattern of the components.

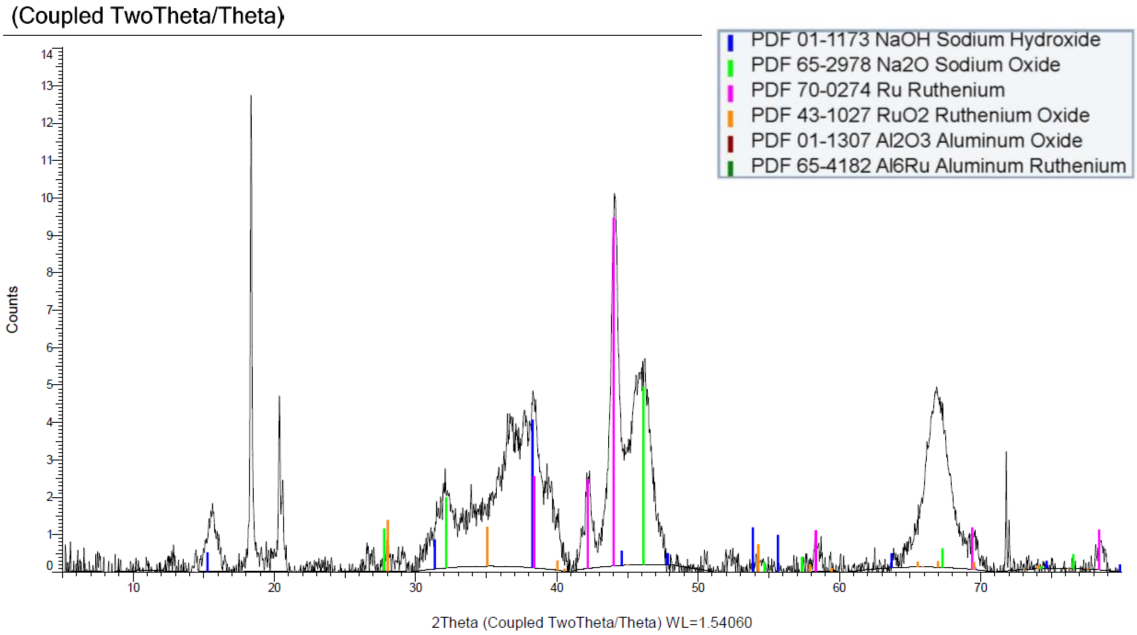

Figure S8. XRD diffractogram of as- synthesised (3%Ru)10%Na/Al_2O_3 and assignment pattern of the components.

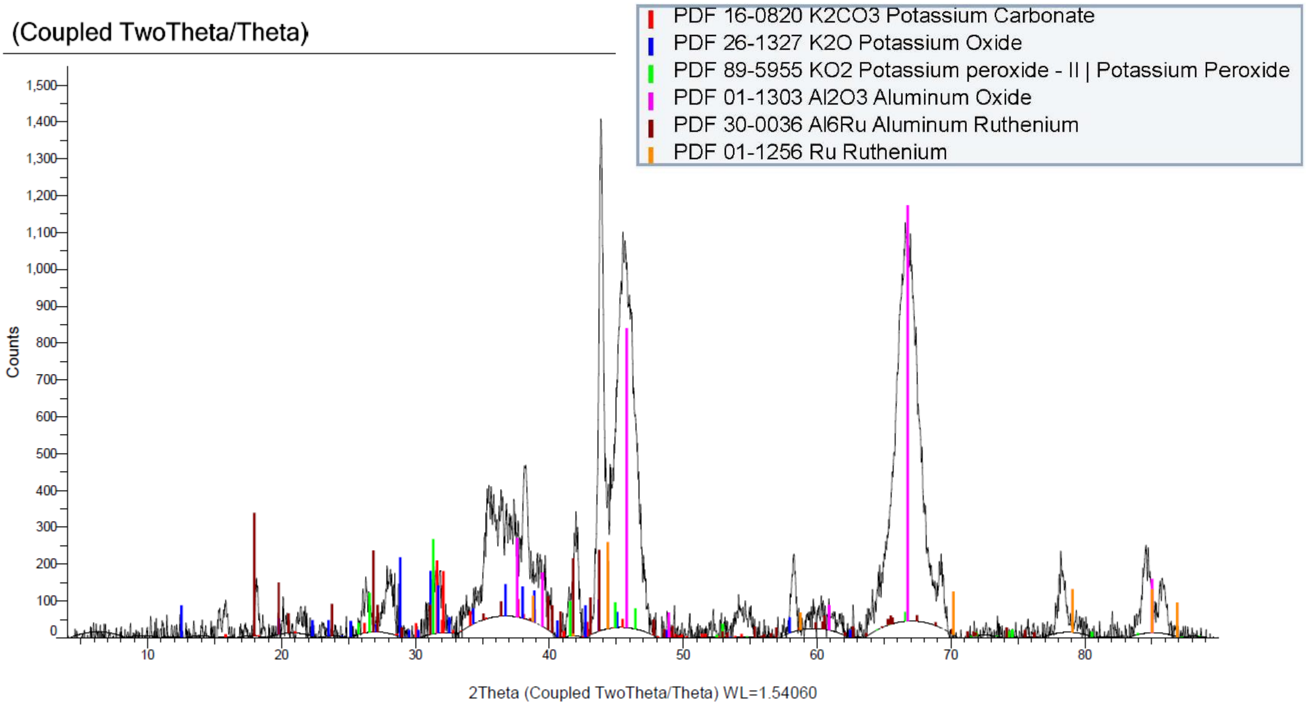

Figure S9. XRD diffractogram of as- synthesised (3%Ru)10%K/Al_2O_3 and assignment pattern of the components.

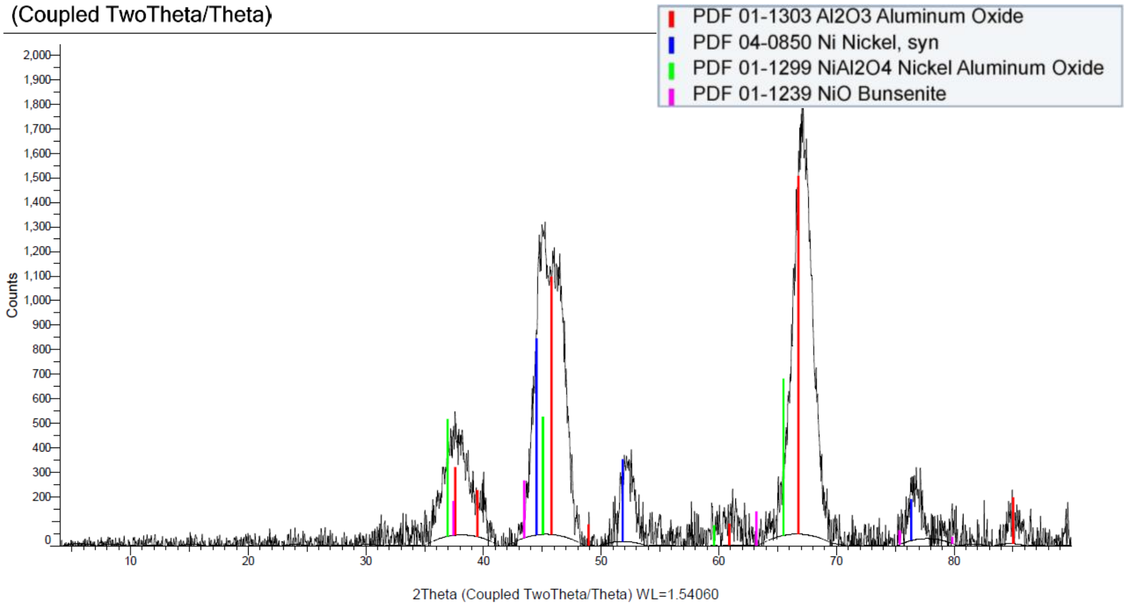

Figure S10. XRD diffractogram of as- synthesised 15%Ni/Al_2O_3 and assignment pattern of the components.

NiBa

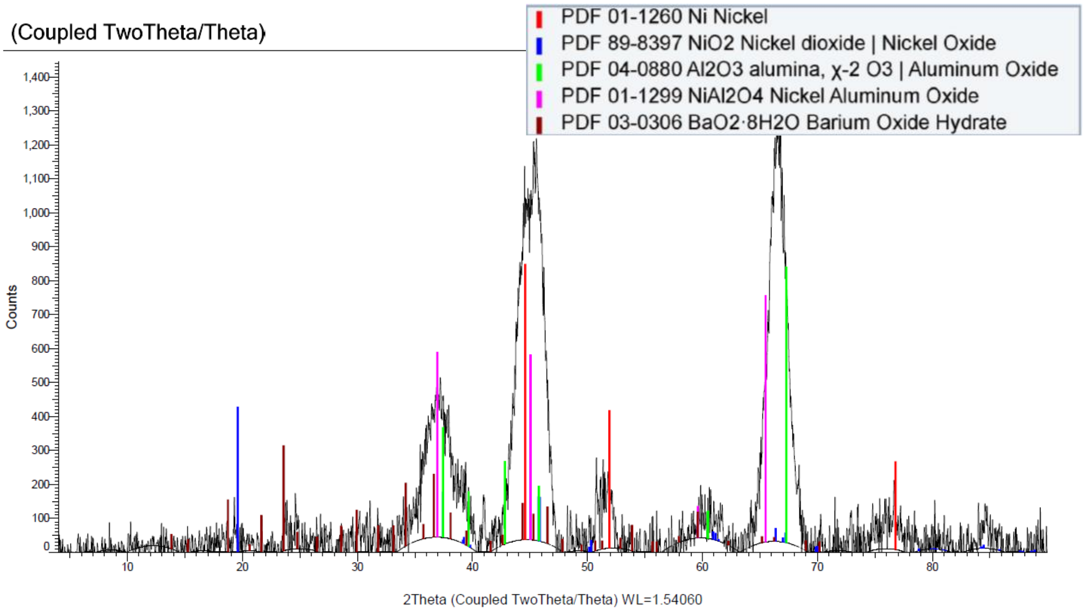

Figure S11. XRD diffractogram of as- synthesised (10%Ba)15%Ni/Al_2O_3 and assignment pattern of the components.

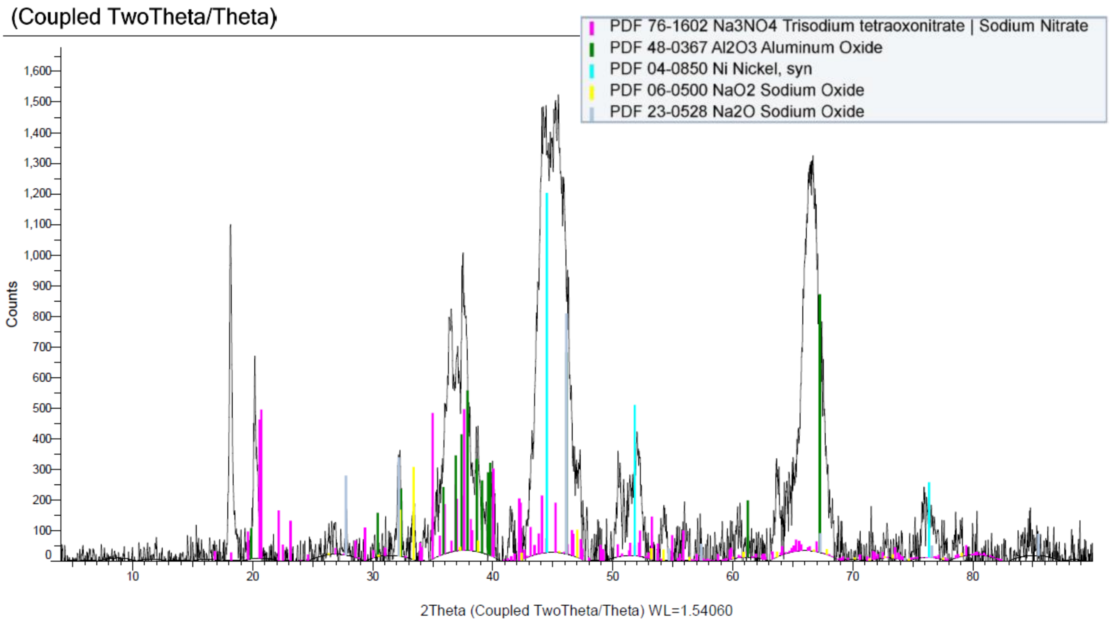

Figure S12. XRD diffractogram of as- synthesised (10%Na)15%Ni/Al_2O_3 and assignment pattern of the components.

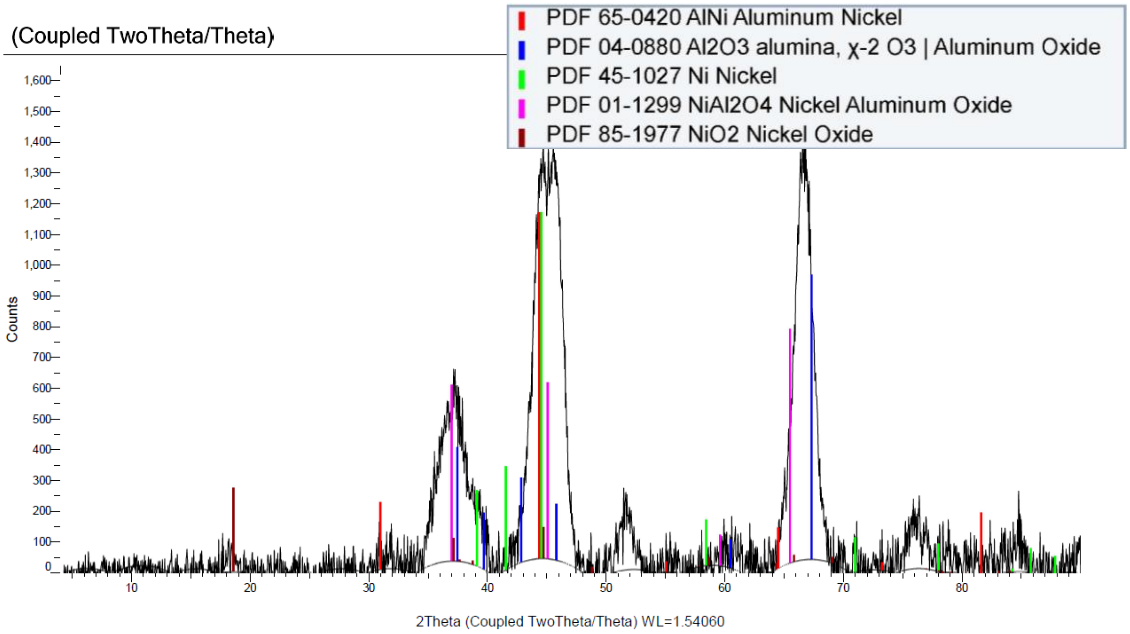

Figure S13. XRD diffractogram of as- synthesised (10%K)15%Ni/Al_2O_3 and assignment pattern of the components.
